# Systematic Process Framework for Conducting Implementation Science Research in Food Fortification Programs

**DOI:** 10.9745/GHSP-D-20-00707

**Published:** 2021-06-30

**Authors:** Emily Teachout, Laura A. Rowe, Helena Pachon, Becky L. Tsang, Lorraine F. Yeung, Jorge Rosenthal, Hilda Razzaghi, Meredith Moore, Dora Panagides, Peiman Milani, Michael J. Cannon

**Affiliations:** a Centers for Disease Control and Prevention, Atlanta, GA, USA.; b Food Fortification Initiative, Atlanta, GA, USA.; c Emory University, Atlanta, GA, USA.; d World Food Programme, Rome, Italy.; e World Food Programme, Nairobi, Kenya.

## Abstract

Food fortification has proven to be an effective approach for preventing micronutrient deficiencies in many settings. Factors that lead to successful fortification programs are well established. However, due to the multisectoral nature of fortification and the added complexities present in many settings, the barriers to success are not always evident and the strategies to address them are not always obvious. We developed a systematic process for identifying and addressing gaps in the implementation of a food fortification program. The framework is composed of 4 phases: (1) connect program theory of change to program implementation; (2) develop an implementation research agenda; (3) conduct implementation research; and (4) analyze findings and develop/disseminate recommendations for next steps. We detail steps in each phase to help guide teams through the process. To our knowledge, this is the first attempt to outline a systematic process for applying implementation science research to food fortification. The development of this framework is intended to promote implementation research in the field of food fortification, thus improving access to and effectiveness of this key public health intervention.

## Abstract (precis)

Many challenges still exist to fully scaling up food fortification in lower resource settings. To address this need, a collective group of experts in the fields of food fortification and implementation science developed a systematic process framework to provide a tool for identifying and working through challenges.

## Key Messages

- There are many countries implementing food fortification programs but not all of them are achieving their public health goals.
- Implementation challenges are best addressed not in an ad hoc way, but rather through a systematic approach known as implementation science.
- The need for implementation science research in public health nutrition programs has been well-recognized.
- Implementation research teams (often composed of scientists, program implementers, and funders) should work together through a systematic process of identifying and addressing gaps in the implementation of a food fortification program.
- This framework provides a systematic way to identify gaps in context-specific programmatic knowledge and action, formulate implementation research questions, prioritize those questions, and supply guidance on how to move forward after the conclusion and analysis of the implementation research studies.

## INTRODUCTION

Food fortification has been demonstrated to be an effective and cost-effective approach for reducing micronutrient deficiencies in many settings where governments have created, implemented, monitored, and enforced standards for the fortification of staple foods.^1^^,^^2^ Factors that lead to successful food fortification programs are well established. Particularly, food fortification programs tend to be more successful when the fortified food vehicle is processed centrally and when a large proportion of the target population consumes this food regularly. Other factors include having appropriate policies and/or standards in place, adequate program coordination across various ministries and agencies, good program monitoring, and proper internal and external quality assurance and quality control.^3^^–^^6^

Sometimes, contextual challenges can impede the successful implementation of food fortification programs.^7^^–^^10^ These challenges can include (but are not limited to) decentralized processing of staple foods, poor regulation and enforcement capacity, or inadequate distribution infrastructure.^9^^,^^11^ Although the best practices for implementing food fortification have been well defined, a process for diagnosing and overcoming obstacles to successful implementation has not. To address this need, we present a systematic process framework that provides a tool for identifying and working through challenges.

Best practices for implementing food fortification have been well defined but a process for diagnosing and overcoming obstacles to successful implementation has not.

Implementation challenges are best addressed not in an ad hoc way, but rather through a systematic approach known as implementation science. The need for implementation science research in public health nutrition programs has been recognized.^11^^–^^18^ Implementation science is the study of methods for improving the execution of programs across varying contexts, with a focus on understanding implementation outcome variables such as acceptability, adoption, appropriateness, coverage, feasibility, fidelity, implementation cost, and sustainability. Implementation research is the application of scientific methods to describe, explore, and explain contextual barriers to implementation; test implementation improvement strategies (innovative strategies to improve the implementation of the intervention); and identify or predict when an implementation improvement strategy might be appropriate for scale.^19^^,^^20^ The systematic process framework we present contributes to the existing literature by interpreting the concepts of implementation science and implementation research as they relate to food fortification. It also provides a systematic way to identify gaps in context-specific programmatic knowledge and action, formulate implementation research questions, prioritize those questions, and supply guidance on how to move forward after the conclusion and analysis of the implementation research studies. To our knowledge, this is the first article that outlines a process for the application of implementation science specific to food fortification and the first to provide a process for systematically identifying and prioritizing implementation research questions for this important public health intervention.

## DEVELOPMENT OF THE FRAMEWORK

Global work in food fortification has identified a need for a diagnostic tool to ascertain gaps in knowledge and action related to developing and applying implementation improvement strategies in the process of translating food fortification programs from high-resource to low-resource settings. To inform our process for applying concepts of implementation science to food fortification, we conducted a targeted review of relevant literature. We included both peer-reviewed and gray literature. We used electronic journal databases such as PubMed and search engines such as Google Scholar. We included English search terms such as “implementation research,” “implementation research framework,” “implementation science,” “implementation science framework,” “implementation research in nutrition,” “implementation research in health programs,” “implementation research in food fortification programs,” and “food fortification.”

We later reviewed literature on commonly used public health programmatic tools such as logic models, theory of change, and the Program Assessment Guide to inform our approaches for developing and prioritizing an implementation research agenda (Phases I and II of the framework).^20^^,^^21^ Our framework uses modified versions of theory of change methodology, a tool that is commonly used in program planning and evaluation. The methodology uses a process of identifying a long-term health goal and mapping programmatic outcomes backward to identify underlying determinants that must be met to achieve the goal. We also use a modified version of the operational research prioritization table from the program assessment guide. This tool provides a system for prioritizing research questions by factors such as cost, time, and relevance.^20^^,^^21^ To evaluate the utility of our model, we applied and refined it while working with partners to develop and execute an implementation research agenda for maize flour fortification in Tanzania. While we specifically designed this framework for food fortification programs in low-resource settings, it may also be applicable in middle- and high-resource settings. This model may also be applied to other complex public health interventions.

## THE FRAMEWORK

**FIGURE 1:**
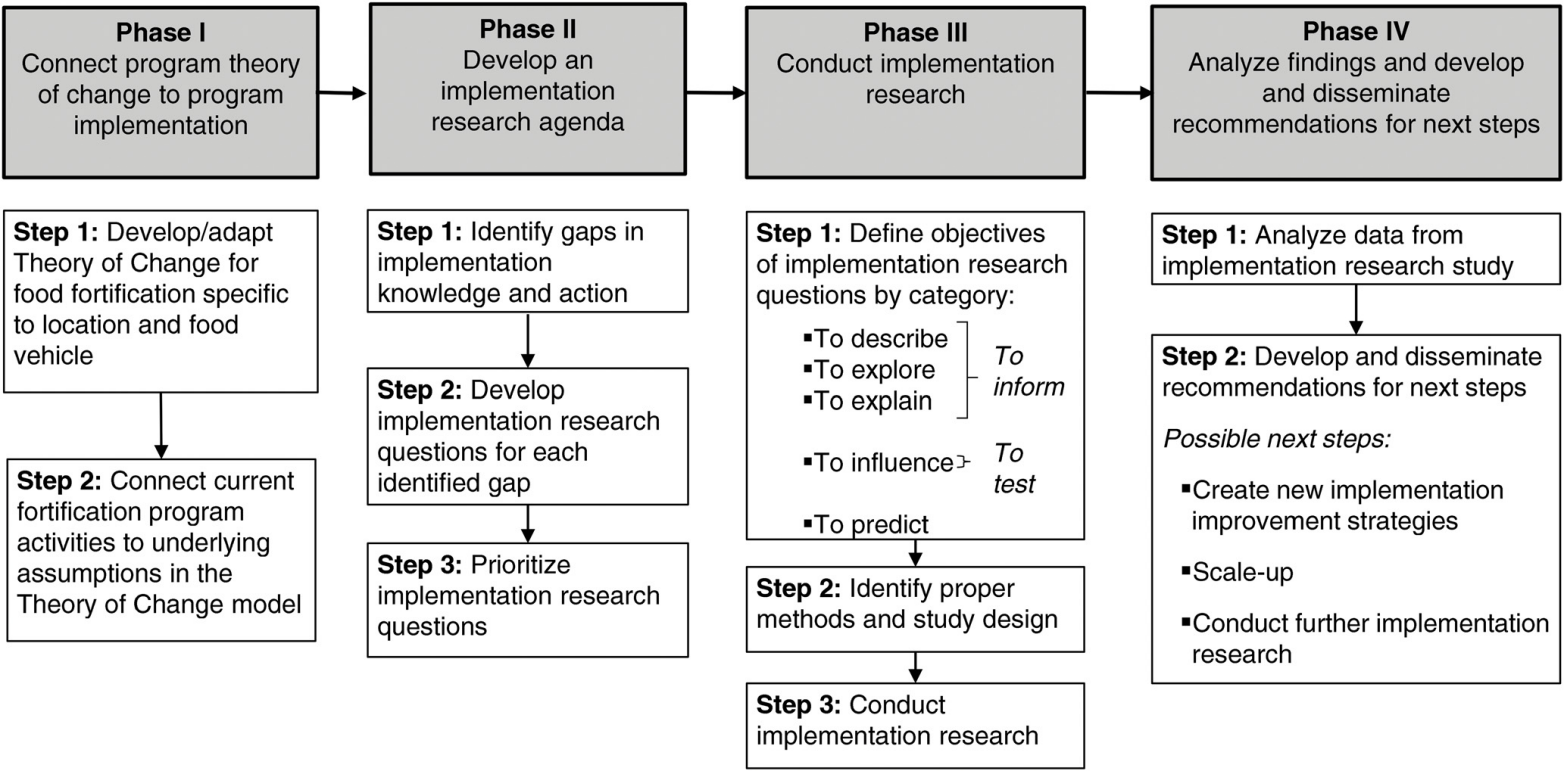
Systematic Process Framework for Conducting Implementation Science Research in Food Fortification Programs

The framework presented here (Figure 1) is designed to guide an implementation research team (often composed of scientists, program implementers, and funders) through a systematic process of identifying and addressing gaps in the implementation of a food fortification program. The framework is composed of 4 phases: (1) connect program theory of change to program implementation; (2) develop an implementation research agenda; (3) conduct implementation research; and (4) analyze findings and develop and disseminate recommendations for next steps. Each phase contains steps to guide teams through the process.

The framework can guide an implementation research team through a systematic process of identifying and addressing gaps in implementing a food fortification program.

### Phase I: Connect Program Theory of Change to Program Implementation

Phase I aims to identify underlying determinants not being met by program activities and generate implementation research questions. Phase I addresses 2 questions that help achieve this goal: (1) What are the underlying determinants that need to be met to implement a successful food fortification program? (2) What program activities and implementation improvement strategies are currently in place to address these determinants?

Successful food fortification programs must provide regular access to adequately fortified foods to a target population; however, the degree to which underlying programmatic determinants are met depends on the context in which a program is being implemented. Food fortification programs are inherently complex due to their multisectoral nature; food fortification programs require buy-in and cooperation from food processors, government legislative and regulatory bodies, and civil service organizations. Additionally, programs in low-resource settings often face added complexities that contribute to the difficulties in meeting underlying programmatic determinants. As a result, barriers to program implementation are not always evident, and strategies to address the barriers are not always obvious.

To identify and address the barriers, an implementation research team must first have a clear understanding of the program theory of change. The team also needs to understand what food fortification activities and implementation improvement strategies are currently occurring so that the gaps between theory and practice can be identified. Because program implementation occurs through the efforts of a wide array of actors from the public and private sectors, the implementation research team will need to carefully consider what stakeholders (in addition to the implementation research team) will need to participate in this phase of the framework.^22^ The group will need to have a broad knowledge of fortification activities that are being implemented by all food fortification stakeholders.

#### Phase I, Step 1: Develop and Adapt Theory of Change for Food Fortification Specific to Location and Food Vehicle

**FIGURE 2:**
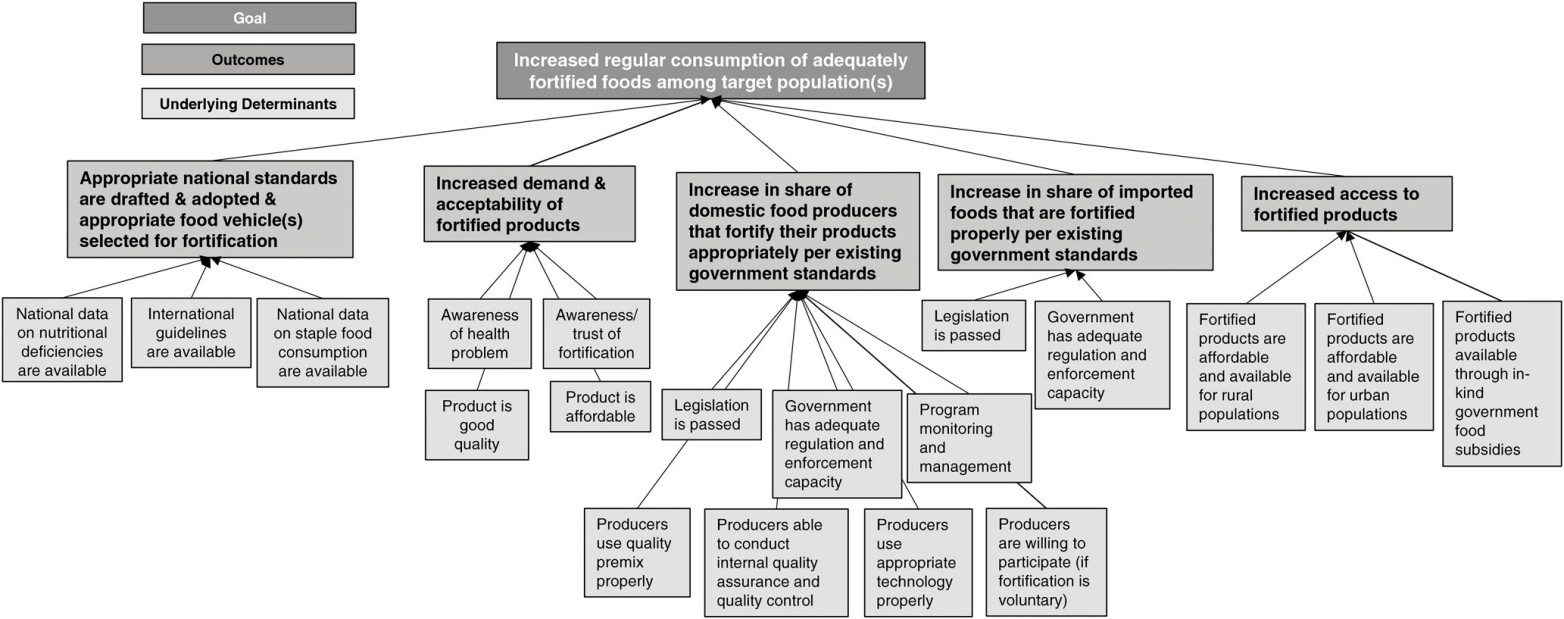
Example of a Theory of Change Model for Food Fortification^a^ ^a^ Created by members of the Global Fortification Technical Advisory Group.

The first step in Phase I is to outline the theory of how the program will succeed by creating a theory of change model. In Figure 2, we provide an example of a theory of change model for food fortification, which should be adapted to the setting and the food vehicles to which it is applied. It should also be adjusted for the type of food fortification being implemented (voluntary, mandatory, etc.), as this may drastically change the implementation strategy. The model was created by the Global Fortification Technical Advisory Group, which is made up of global food fortification subject matter experts. This model was developed by ensuring that implementation outcome variables (Table 1) informed the immediate outcomes in the model.^20^ This model illustrates a pathway that may lead to the increased regular consumption of adequately fortified food in a target population.

**TABLE 1.** Implementation Outcome Variables Contextualized for Food Fortification^19^

Implementation Outcome Variable	Working Definition in the Context of Food Fortification	Related Terms in Food Fortification (Non-exhaustive)	Examples of Interpretations for Food Fortification Programs (Non-exhaustive)
Acceptability	The degree to which food fortification (or an innovation that addresses a precondition to the increased consumption of adequately fortified foods) is agreeable to stakeholders	Perceived operability; myths, taboos, and beliefs; perceived cost- benefit; perceived affordability; perceived business advantage or disadvantage; organoleptic/sensory properties	Acceptability of food fortification technology by industry; acceptability of the intervention by policy makers; acceptability of the addition of micronutrients to food by consumers; acceptability of a price increase by consumers
Adoption	The uptake, utilization, intention to try or intention to consume any product, activity, or innovation related to food fortification	Uptake; penetration; utilization; intention to try or intention to buy/consume	Adoption of food fortification by producers; adoption of a fortification logo; use or intended use of fortified products by consumers
Appropriateness/ Feasibility	The perceived (or actual) fit, relevance, or compatibility of food fortification for a particular context; the ability of food fortification to reach a particular target group (e.g., women of reproductive age who are below the poverty line)	Practicality; perceived fit; relevance; suitability; operability	Appropriateness of the food selected to fortify; appropriateness of the micronutrient compound being used by food processors; feasibility of fortification in the context of the state of the production industry (e.g., centralized vs decentralized processing); appropriateness of the technology being used by producers; appropriateness of the intervention to address a particular micronutrient deficiency; feasibility to enforce legislation
Coverage	The degree to which the targeted population is consuming fortified products regularly or able to access fortified products	Reach; access; penetration; consumption amongst the population; coverage of the target population	Penetration of fortified products in the market; percentage of the population that can access fortified products
Fidelity	The degree to which activities related to food fortification are occurring according to original program plan, policy, or protocols	Compliance; adherence; quality; delivery as intended	Compliance of the micronutrient compound with national standards; compliance of micronutrient levels found in foods with allowable ranges that are specified by national standards
Implementation Cost	The total cost of implementing all activities related to achieving increased consumption of adequately fortified foods	Program cost	Total cost per beneficiary of reaching 80% coverage of fortified foods
Sustainability	The extent to which fortification is institutionalized within the government and industry and not reliant on continuous external inputs and support	Institutionalization; integration; non-reliance on external inputs; ownership by the government, industry, and society	Degree to which the government can monitor and enforce food fortification; degree to which complying with fortification requirements is a viable business model for producers

#### Phase I, Step 2: Connect Current Fortification Program Activities and Implementation Improvement Strategies to Determinants in the Theory of Change Model

**FIGURE 3:**
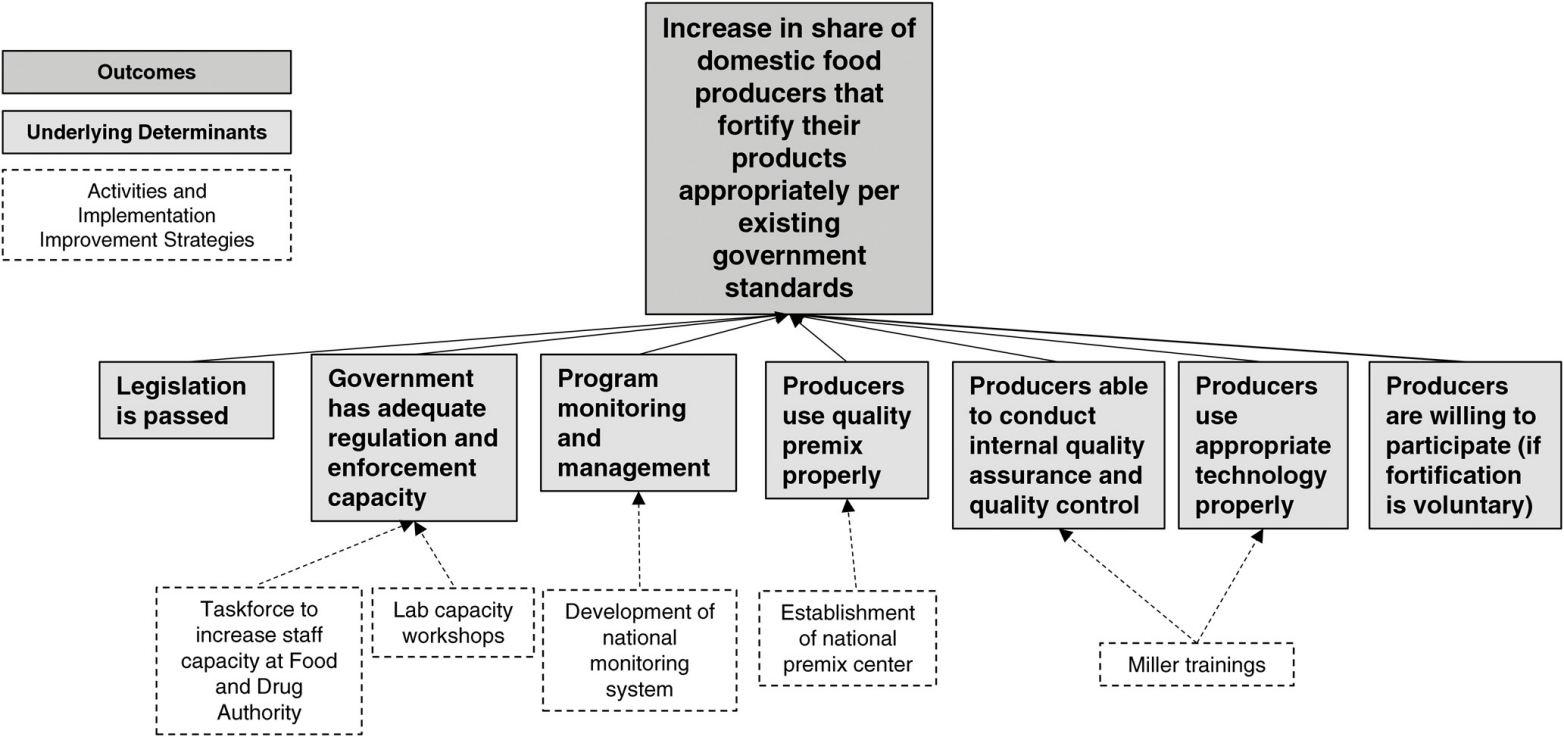
Example of Phase I, Step 2 of Food Fortification Implementation Framework

The second step in Phase I is to identify how the current program activities and implementation improvement strategies (dotted boxes in Figure 3) address underlying determinants (light gray boxes in Figure 2). To do this, the implementation research team leads the stakeholders in a brainstorming activity to develop an exhaustive list of current activities and implementation improvement strategies relevant to food fortification. Then, the group connects each activity and implementation improvement strategy to any determinants that they might address in the theory of change model. This step repeats for each activity in the list. It may be useful to draw lines between activities and theory of change determinants. Figure 3 shows an example of how some implementation improvement strategies can be connected to the theory of change model.

### Phase II: Develop an Implementation Research Agenda

Phase II is composed of 3 steps: identification of programmatic gaps, research-question generation, and question prioritization. This phase takes an implementation research team through a systematic process of looking at all the possible gaps between the program implementation and the theory of change model for achieving the overall programmatic goal. By identifying all the gaps, the team can identify and prioritize pertinent research questions that affect implementation outcome variables such as acceptability, adoption, appropriateness, coverage, feasibility, fidelity, implementation cost, and sustainability (Table 1).

By identifying gaps, the team can identify and prioritize pertinent research questions that affect implementation outcome variables.

#### Phase II, Step 1: Identify Gaps in Implementation Knowledge and Action

To identify gaps in the implementation of a food fortification program, the team participating in Phase II should examine the ongoing program implementation activities and implementation improvement strategies that have been mapped onto the determinants in the theory of change model. After connecting the exhaustive list of strategies with the particular determinants (in Phase I), it will be evident where some of the gaps are. Perhaps the program does not address some of the determinants that the team had identified as necessary for successful fortification implementation outcomes, or perhaps the program includes some activities or strategies that they were unable to connect, and thus might not be necessary. To identify gaps that are not immediately evident, the team should go through each determinant and ask the questions that we have developed in the Box.

BOX **Questions to Answer for Each Assumption/Determinant in the Theory of Change Model**

- Are there any other ongoing activities or implementation improvement strategies that address this assumption/determinant?
- Which ongoing activities and implementation improvement strategies do we know are working well? How do we know?
- Which ongoing activities do we know are not working well? How do we know?
- Which ongoing activities do not have enough monitoring and evaluation data to determine if they are working well?
- Are the ongoing activities or implementation improvement strategies sufficient to address each assumption/determinant?
- What preconditions are not addressed through an ongoing activity or implementation improvement strategy? Do we have enough knowledge to develop an implementation improvement strategy to address that assumption/determinant? If not, what do we need to know before we could develop an implementation improvement strategy? What are the consequences if a particular determinant is not addressed?

#### Phase II, Step 2: Develop Implementation Research Questions for Each Identified Gap

The next step is to decide whether each identified gap is a gap in knowledge, action, or both. This will help determine what the research questions should be. For example, if the implementation research team has identified that there is no activity to address the precondition, “producers use quality premix appropriately,” and the team has determined that there is not enough knowledge regarding whether processors are using quality premix appropriately or how to influence that behavior, then there are gaps in knowledge. The resulting research questions might be: “what proportion of producers in the program region are using quality premix appropriately?” and “what are the factors that influence whether a processor uses quality premix appropriately?” Alternatively, if there is not an activity to address that precondition, and the team has determined that there is enough knowledge to determine that millers are not using premix appropriately, then there are gaps in action. If the team decides to develop and test an implementation improvement strategy to fill this gap, an example of a research question might be: “what is the impact of a 3-day intensive miller training on using quality premix appropriately?”

#### Phase II, Step 3: Prioritize Implementation Research Questions

This previous step may result in a long list of research questions. Because there may be limited resources to conduct implementation research and not all questions will be equally important to address immediately, the team should prioritize the questions. To do this, the team should use criteria such as cost of answering the research question, time investment, timeliness, and importance for programmatic success. We have provided a template (Table 2) for the prioritization of implementation research questions, modeled on an approach found in the program assessment guide.^21^

**TABLE 2.** Template for Prioritizing Implementation Research Questions^21^

	Example
Program outcome and underlying assumption/determinant	Increased access to fortified products. Fortified products are affordable and available for urban populations
Research question(s)	What is the national coverage of fortified products amongst the urban poor?
How will the success of the intervention be affected by the information provided?	The urban poor are a large proportion of our target population. If we are not currently reaching them, we will need to adjust our program with new targeting strategies.
Possible research methods	Secondary analysis of existing data set
Organization/persons responsible for conducting the research	The National Micronutrient Committee
Resources, support, or training required by the organization/persons	Human resources to analyze data and write report
Estimated cost	No additional cost to program
Estimated time to prepare for and conduct the research	6–8 weeks

### Phase III: Conduct Implementation Research

Phase III involves defining the objectives of each implementation research question, identifying methods and study designs, and conducting the research. As with all research, the extent to which the data will provide useful information depends on the level of scientific rigor used when designing, conducting, and analyzing the study.

#### Phase III, Step 1: Define Objectives of Implementation Research Questions by Category

Implementation research questions can be categorized into 1 or more standardized objectives (to describe, to explore, to explain, to influence, to predict) described in the World Health Organization's (WHO) *Implementation Research in Health: A Practical Guide* (Table 3).^19^^,^^23^ Understanding the objective of a particular research question can help the team to refine the appropriate methods and inform the next steps after data analysis. If the research objective is to describe, explore, or explain the underlying determinants, then the research is designed to inform potential implementation improvement strategies. If the objective is to influence determinants, then the research is designed to test an implementation improvement strategy to see if it has the desired effects on the implementation outcome variable. If the objective is to predict, then the research is designed to forecast the likely success of scaling-up implementation improvement strategies.

**TABLE 3.** Implementation Research Objectives With Examples for Food Fortification Programs^a^

Research Objective	Description	Examples
To describe	Describes the context in which food fortification is occurring and the main components that may affect the success of food fortification	What proportion of the population consumes wheat flour that is produced in industrial mills?
To explore	Explores the possible barriers and facilitators to the implementation of food fortification	What are the barriers and facilitators for implementing adequate external quality assurance/quality control?
To explain	Explains how and why certain aspects of the intervention or the context may influence implementation outcomes	How do market prices affect revenue margins for wheat flour millers?
To influence	Tests desired effects of an implementation improvement strategy	Does additional training and fortification sensitization of millers lead to better fortification of a product?
To predict	Predicts whether the same (or modified version of) aspects of a food fortification program will work under various conditions (useful for scale-up)	Will the radio advocacy materials that increased fortification logo awareness in the southern parts of the country also produce similar results in the northern parts of the country?

a Adapted from Peters DH, Tran NT, Taghreed A. *Implementation Research in Health: A Practical Guide*. World Health Organization; 2013.

#### Phase III, Step 2: Identify Proper Methods and Study Design

Implementation research does not require a unique set of methods, which could include surveys, focus group discussions, participatory action research, scenario-building exercises, economic modeling, or a variety of others that are listed in the WHO manual for implementation research.^19^ The choice of methods will depend on the research question(s) being asked and the level of confidence desired.

#### Phase III, Step 3: Conduct Implementation Research

How to conduct research is beyond the scope of this article. However, conducting implementation research does not require unique considerations. For example, the implementation research team may need to ensure that institutional review board approval has been obtained, research tools have been developed and piloted, a field team has been trained properly, and logistics for data collection and analysis have been planned.

### Phase IV: Analyze Findings and Develop and Disseminate Recommendations for Next Steps

The final phase in the framework involves data analysis, interpretation of findings, and the development and dissemination of recommendations. Developing and disseminating recommendations through appropriate channels is paramount for making implementation research useful. Some appropriate channels might include national fortification alliances, working groups, academic journals, government meetings, and conferences. Recommendations are essential for program implementers and they expand the knowledge base in a way that is valuable for other researchers and potential funders.^13^

Developing and disseminating recommendations through appropriate channels is paramount for making implementation research useful.

#### Phase IV, Step 1: Analyze Data From Implementation Research Study

The choice of data analysis techniques will depend on the research methods employed. Data analysis and dissemination must accommodate the time constraints of the program implementers and funders. These decision makers may have specific deadlines for deciding on program scale-up or programmatic activities for the next funding period; if the data cannot be analyzed in a practical timeframe, the implementation research may not be worth conducting.^15^

#### Phase IV, Step 2: Develop and Disseminate Recommendations for Next Steps

**FIGURE 4:**
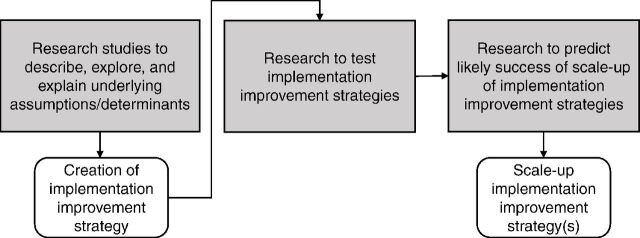
Possible Next Steps After the Conclusion of an Implementation Research Study

The recommendations for next steps differ depending on whether the purpose of the implementation research question(s) was to describe, explore, explain, test, or predict (Figure 4). If the research intends to inform implementation improvement strategies by addressing a gap in knowledge through a descriptive, exploratory, or explanatory study, the recommendation might be to create and test a new implementation improvement strategy. If the research tested an implementation improvement strategy, the recommendation would depend on whether the strategy was successful or not. If it was successful, the recommendation might be to identify the likelihood of success of the scale-up of the implementation improvement strategy; if it was not successful, the recommendation might be to conduct new research to inform the development of new implementation improvement strategies. If the objective of the research was to predict the likely success of the scale-up of an implementation improvement strategy in various settings, the recommendation would relate to whether or how to scale up an implementation improvement strategy.

## CONCLUSION

There are many countries implementing food fortification programs but not all of them are achieving their public health goals. In many cases, this is due to underlying contextual determinants that affect the ability of the program to succeed. In this article, we have introduced a systematic process for how to identify key gaps in implementation, develop and prioritize implementation research questions, and carry out an implementation research agenda that will inform implementation improvement strategies. The process framework we present emphasizes the importance of identifying and prioritizing research questions in a systematic way that includes partners from both the public and private sector who are involved in the implementation of various food fortification activities (policy, food production, regulation, and enforcement, etc.). Our framework assumes that food fortification is already occurring; it is a process for identifying and studying gaps in ongoing implementation. Additionally, the process that we present assumes the need for a facilitating organization or person to bring together fortification partners from the public and private sectors. The development of this framework is intended to promote implementation research in the field of food fortification and thus improve implementation outcomes of this key public health intervention, especially in low-resource settings.

## Notes

**Peer Reviewed**

**First published online:** May 27, 2021.
